# Morphological, compositional, structural, and optical properties of Si-nc embedded in SiO_*x*_ films

**DOI:** 10.1186/1556-276X-7-604

**Published:** 2012-10-30

**Authors:** J Alberto Luna López, J Carrillo López, D E Vázquez Valerdi, G García Salgado, T Díaz-Becerril, A Ponce Pedraza, F J Flores Gracia

**Affiliations:** 1 IC-CIDS Benemérita Universidad Autónoma de Puebla, Ed. 103 C o D, Col. San Manuel, C.P, Puebla, Pue, 72570, Mexico; 2 Department of Physics & Astronomy, University of Texas at San Antonio, San Antonio, TX, 78249, USA

**Keywords:** Silicon nanocrystals, High-resolution TEM, XRD, PL, AFM, HFCVD, 61.05.-a, 68.37.Og, 61.05.cp, 78.55.-m, 68.37.Ps, 81.15.Gh

## Abstract

Structural, compositional, morphological, and optical properties of silicon nanocrystal (Si-nc) embedded in a matrix of non-stoichiometric silicon oxide (SiO_*x*_) films were studied. SiO_*x*_ films were prepared by hot filament chemical vapor deposition technique in the 900 to 1,400°C range. Different microscopic and spectroscopic characterization techniques were used. The film composition changes with the growth temperature as Fourier transform infrared spectroscopy, energy dispersive X-ray spectroscopy, and X-ray photoelectron spectroscopy reveal. High-resolution transmission electron microscopy supports the existence of Si-ncs with a diameter from 1 to 6.5 nm in the matrix of SiO_*x*_ films. The films emit in a wide photoluminescent spectrum, and the maximum peak emission shows a blueshift as the growth temperature decreases. On the other hand, transmittance spectra showed a wavelength shift of the absorption border, indicating an increase in the energy optical bandgap, when the growth temperature decreases. A relationship between composition, Si-nc size, energy bandgap, PL, and surface morphology was obtained. According to these results, we have analyzed the dependence of PL on the composition, structure, and morphology of the Si-ncs embedded in a matrix of non-stoichiometric SiO_*x*_ films.

## Background

Since the discovery of light emission from porous silicon [1], an intense investigation of materials compatible with silicon technology with excellent optical properties has been under development. Recently, materials containing silicon nanocrystal (Si-nc) have attracted the interest of researchers due to their optical properties. Therefore, a great variety of materials with these characteristics have been studied [2-5]. One of these materials is the non-stoichiometric silicon oxide (SiO_*x*_); this material contains Si excess agglomerates to create Si nanoparticles embedded in an oxide matrix. SiO_*x*_ shows some special compositional, structural, morphological, and optical properties that vary with the Si excess. In particular, the optical characteristics of SiO_*x*_ films can be varied with the growth temperatures. For example, refractive index varies from 1.6 to 2.4 when the growth temperature is changed; also, SiO_*x*_ emits visible light. These characteristics have given place to various types of applications such as waveguides, no volatile memories, light radiation, and detection devices [6-8]. Furthermore, the fabrication of SiO_*x*_ films is completely compatible with complementary metal oxide semiconductor technology, providing an easy way for the optoelectronic integration on silicon. Several techniques have been employed to make thin SiO_*x*_ films, such as high-dose Si ion implantation into SiO_2_ films [6,9], low pressure chemical vapor deposition [8], sol–gel, hot filament chemical vapor deposition (HFCVD) [10,11], and plasma enhanced chemical vapor deposition [12], in which an improvement of the optical and structural properties as the PL emission and Si excess has been reported. In this work, HFCVD technique was used to deposit SiO_*x*_ films on silicon and quartz substrates. This technique allows us to obtain thin SiO_*x*_ films with different properties just by varying the source-substrate distance during the deposit; this distance changes the growth temperature. Structural, compositional, morphological, and optical properties of SiO_*x*_ films prepared by HFCVD and an analysis of the composition, morphology, Si-nc size, and their relation with the PL emission are presented.

## Methods

Si-ncs embedded in thin SiO_*x*_ films were deposited on quartz and n-type silicon (100) substrates, the silicon substrates with 2,000- to 5,000-Ω cm resistivity in a horizontal hot filament CVD reactor using quartz and porous silicon as the sources. A hot filament at approximately 2,000°C dissociates ultra-high purity molecular hydrogen which flows into the reactor at a 50 sccm rate and produces atomic hydrogen (H). Something worth mentioning in this process is the use of a hydrogen flux on top of the hot filament, which resulted in a remarkable improvement of the optical and structural properties of the SiO_*x*_ films deposited. The substrates were carefully cleaned with a metal oxide semiconductor standard cleaning process, and the native oxide was removed with an HF buffer solution before being introduced into the reactor. The heating rate depends on the source-substrate distance (dss). The reactive species (H) forms a volatile precursor (SiO) deposited on the silicon substrate and produces Si-ncs embedded in thin SiO_*x*_ films. The filament-source distance was kept constant (2 mm). The relationship between the filament temperature (approximately 2,000°C) and the variation of the dss of 2, 3, 4, 5, and 6 mm provides a change in the growth temperature (Tg) of 1,400°C, 1,300°C, 1,150°C, 1,050°C, and 900°C, which was measured with a thermocouple in each position, respectively. These changes in the dss and Tg, consequently, have modified the silicon excess and defects in the non-stoichiometric SiO_*x*_ films. The film refractive index and the film thickness were measured using a null Ellipsometer Gaertner L117 (Gaertner Scientific Co., Chicago, IL, USA) with a laser of He-Ne (632.8 nm); the film thickness was measured using a Dektak 150 profilometer (Veeco Instruments Inc., Plainview, NY, USA). FTIR spectroscopy measurements were done using a Bruker system model vector 22 (Bruker Instruments, Bellirica, MA, USA). XPS analysis was carried out using a Thermo Fisher spectrometer (Thermo Fisher Scientific, Waltham, MA, USA) with a monochromatic Al radiation XR15 and energy of 15 eV. PL response was measured at room temperature using a Horiba Jobin Yvon spectrometer model FluroMax 3 (Edison, NJ, USA) with a pulsed xenon source whose detector has a multiplier tube, which is controlled by computer. The samples were excited using a 250-nm radiation, and the PL response was recorded between 400 and 900 nm with a resolution of 1 nm. Room-temperature transmittance of the SiO_*x*_ films was measured using a UV–vis-NIR Cary 5000 system (Agilent Technologies Inc., Santa Clara, CA, USA). The transmittance signal was collected from 190 to 1,000 nm with a resolution of 0.5 nm. HRTEM measurements and XEDS were done using a Titan 80- to 300-kV model with an energy spread of 0.8 eV. HRTEM micrographs were analyzed using Gatan DigitalMicrograph software (Gatan Inc. Pleasanton, CA, USA) [13]. The surface morphology of non-stoichiometric SiO_*x*_ films was studied using a scanning probe microscopy of Ambios Technology (Santa Cruz, CA, USA), operated in non-contact mode. A 4 × 4-μm^2^ scanned area was used for each topographic image, and a 460-μm-long single-crystal Si n-type cantilever operated at 12 kHz (type MikroMash SPM Probes (San Jose, CA, USA)) was used. Four different scans were done for each sample, showing good reproducibility. AFM images were analyzed using scanning probe image processor software [14].

## Results and discussion

**Figure 1:**
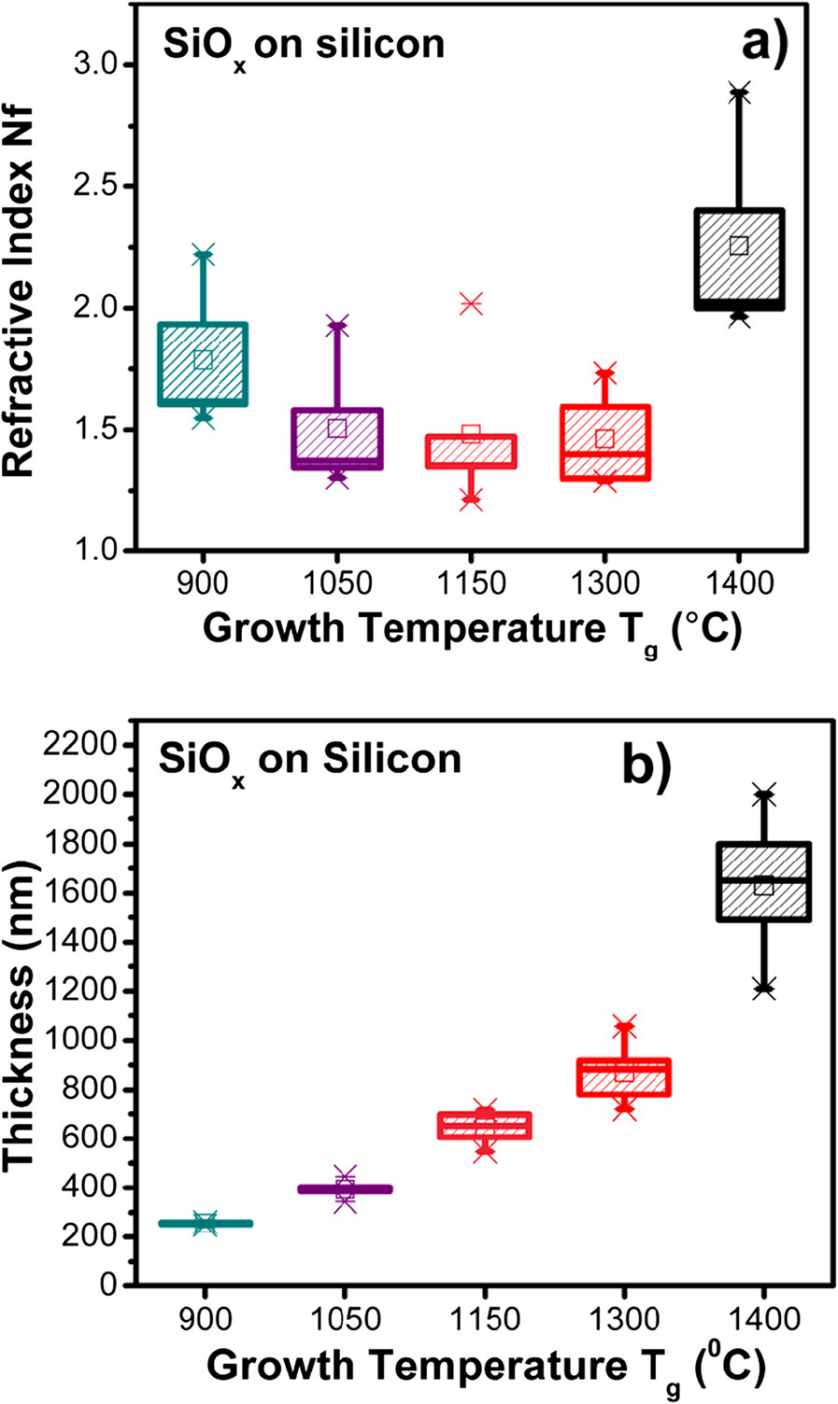
**Refractive index (a) and thickness (b) as functions of growth temperature of the SiO**_***x***_**films**.

The refractive index and thickness of the SiO_*x*_ films as a function of the growth temperature are shown in Figure 1; the refractive index of the thin SiO_*x*_ films changes with Tg. Thicker samples were obtained when the growth temperature was increased from 900°C to 1,400°C. A variation in the refractive index from 1.4 to 2.2 was measured when the growth temperature was increased from 1,150°C to 1,400°C. This variation has been related to a change of the silicon excess in SiO_*x*_ films [12]. Therefore, we can modify the silicon excess in the SiO_*x*_ films by changing the growth temperature.

**Figure 2:**
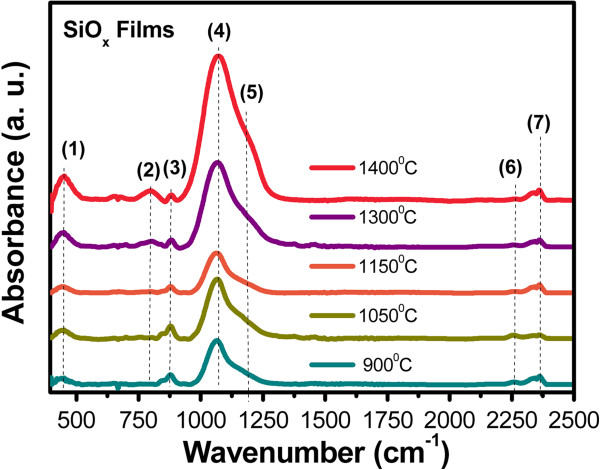
**FTIR absorption spectra of the thin SiO**_***x***_**films for different growth temperature.** The numbers (1 to 7) mean different vibration modes, which are described in Table 1.

FTIR absorption spectra of thin SiO_*x*_ films are shown in Figure 2. These spectra show the absorption peaks associated with the rocking (458 cm^−1^) (peak 1), bending (812 cm^−1^) (peak 2), on-phase stretching (1,084 cm^−1^) (peak 4), and out-of-phase stretching (1,150 cm^−1^) (peak 5) vibration modes of the Si-O-Si bonds in SiO_2_[7,15,16]. The position of the stretching absorption peak in SiO_*x*_ films changes with the growth temperature. Tg produces changes only with regard to the microstructure of the films, where the radiative defects can be activated during the process. The on-phase stretching peak position slightly moves towards a higher wavenumber with a higher growth temperature. On the other hand, the out-of-phase stretching peak position shows similar changes with the growth temperature. The position of peak (4) depends on silicon excess. This is an evidence that the SiO_*x*_ films have a great content of sub-stoichiometric SiO_*x*_ (*x* < 2) phase in the as-deposited state. Peaks in the spectra at 883 cm^−1^ (peak 3), corresponding to Si-H bending and Si-OH, and the other one located at 2,257 cm^−1^ (peak 6), corresponding to Si-H stretching, are observed [17-19]; these bonds are present in the films due to hydrogen incorporation during the growth process. Also, a peak centered at 2,352 cm^−1^ (peak 7) comes from the CO_2_ content in the atmosphere [20]. Furthermore, the peak intensity changes with the growth temperature as shown in Figure 2. A relation between peak intensity and thickness is established; to bigger peak intensity, a bigger thickness. In Figure 1, we can see that if there is a high growth temperature then the thickness increases. Therefore, the peak intensity is bigger too. Moreover, the oxygen and hydrogen contents change with the growth temperature. The hydrogen and oxygen contents decrease when the growth temperature increases, as can be seen with the behavior of the peaks 3 and 7 and peaks 2, 4, and 5, respectively, as shown in Figure 2.

**Figure 3:**
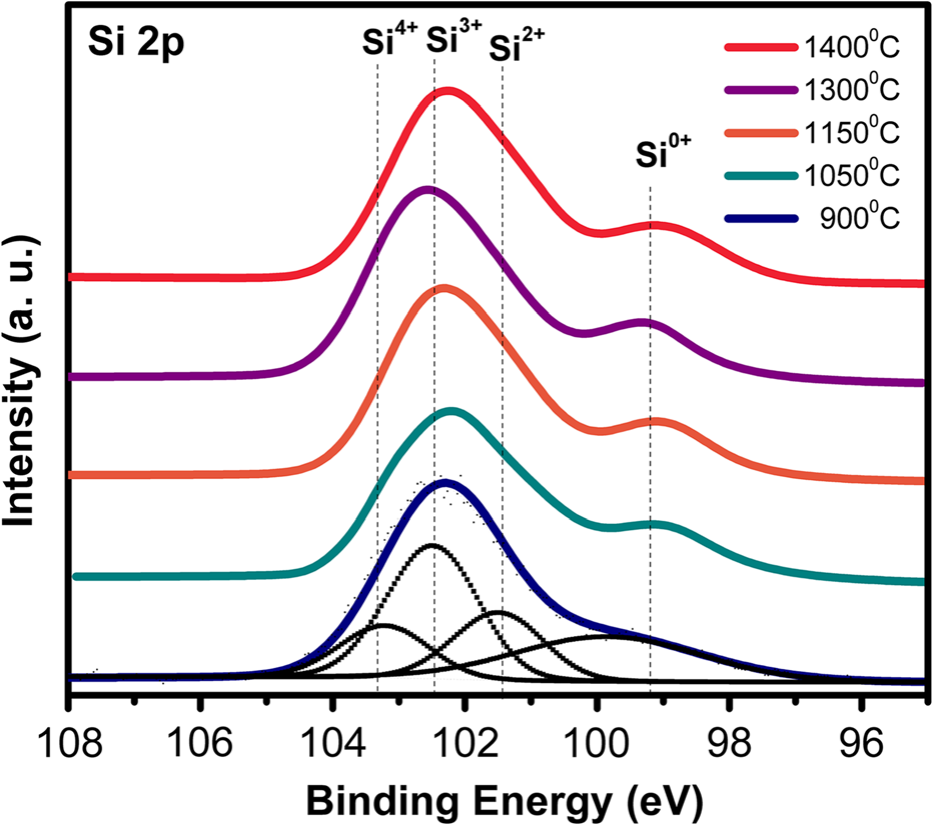
**Si 2p XPS spectra show the composition of the SiO**_***x***_**films.**

XEDS spectra of the SiO_*x*_ films were realized for several growth temperatures; the stoichiometric ratio is determined by the atomic percentages of silicon and oxygen. The peak intensities of oxygen and silicon change with the growth temperature. The peak intensities of silicon are higher when decreasing the growth temperature, and the peak intensities of oxygen decrease when increasing the growth temperature. These variations indicated that the stoichiometry of the SiO_*x*_ films changes with the growth temperature. We can see that the oxygen content decreases with the increase of the growth temperature, and the silicon content decreases with the increase of the growth temperature. Then, with higher growth temperature, the silicon content increases and the oxygen content decreases. In Table 1, the composition results of the XEDS spectra are listed. Figure 3 shows the XPS experimental spectra of the Si 2p line and the evolution of the Si 2p line of different SiO_*x*_ films. The four oxidation states, as well as the unoxidized state, can be modeled as tetrahedral bonding units, in which a central Si atom is bonded to (4 − *n*) Si atoms and *n* oxygen atoms (Si-Si_4 − *n*_O_*n*_) with *n* = 0 to 4. Therefore, the 99.5 eV peak is associated with elemental silicon. SiO_2_ spectra increase the peak energy to 103.3 eV, corresponding to *n* = 4. The Si 2p binding energies are normally about 99 to 103 eV. It is widely accepted that the Si 2p photoelectron peak of SiO_*x*_ contains five components, corresponding to a non-oxidized state and four different oxidation states of Si [21,22]. The variation of the oxidation states of the SiO_*x*_ films leads to peak position’s shift, as shown in Table 2. A peak at about 99 eV accompanied by a peak at about 103 eV is present; they can be attributed to Si and SiO_2_, respectively, and any variation could be attributed to sub-oxidized silicon [23,24]. The increasing electro-negativity of the Si-O bound relative to the Si-Si bond results in a shift to a higher binding energy of the core level electrons in the silicon.

**Table 1:** Compositional results (atomic percentages of oxygen (O) and silicon (Si))

**Temperature (°C)**	**dss (mm)**	**Atomic percentage**		***x*** **= O/Si**
		**O**	**Si**	
*1,400*	2	30.76	69.22	0.44
*1,300*	3	61.60	38.38	1.60
*1,150*	4	56.02	43.96	1.27
*1,050*	5	64.48	35.50	1.81
*900*	6	62.49	37.50	1.66

Results of the SiO_*x*_ films obtained by XEDS.

**Table 2:** **Oxidation states of the SiO**_***x***_**films obtained by means of the convolution of the XPS curves**

		**Oxidation states**			
**Temperature (°C)**	**dss (mm)**	**Peak position (eV)**			
		**Si**^**0+**^	**Si**^**2+**^	**Si**^**3+**^	**Si**^**4+**^
*1,400*	2	99.08	101.06	102.16	102.95
*1,300*	3	99.67		101.98	103.01
*1,150*	4	99.08	101.47	102.51	103.24
*1,050*	5	99.02	101.32	102.28	103.16
*900*	6	99.90	101.36	102.01	103.34

**Figure 4:**
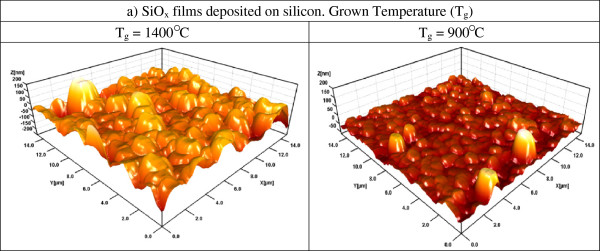
**3D AFM images of SiO**_***x***_**films deposited on silicon substrate at different Tg.** Scanned area is 4 × 4 μm^2^.

**Figure 5:**
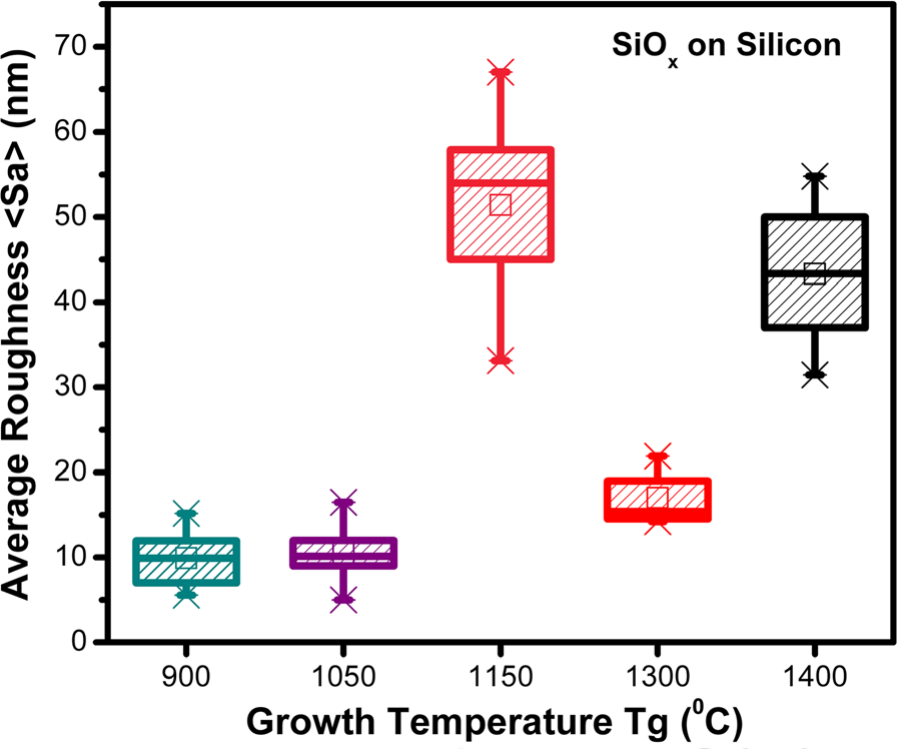
**Average roughness (*S***_**a**_**) as a function of Tg for SiO**_***x***_**films.** Scanned area is 4 × 4 μm^2^.

AFM images of the SiO_*x*_ films in Figure 4 are presented. All images exhibit a rough surface. It can be seen that the surface exhibits different characteristics depending on the growth temperature, which influences the size of the grains (roughness), their form, and composition. Average roughness decreases by decreasing the Tg and thickness [8]. The roughness analysis is shown in Figure 5. It is observed that the surface roughness of SiO_*x*_ films with lower Tg is less than that with higher Tg, except for Tg = 1,150°C. The high roughness of the sample grown at 1,400°C is probably the cause of the index variation with a not clear tendency, and the roughness could be due to the big nanocrystals embedded in the SiO_*x*_ films.

**Figure 6:**
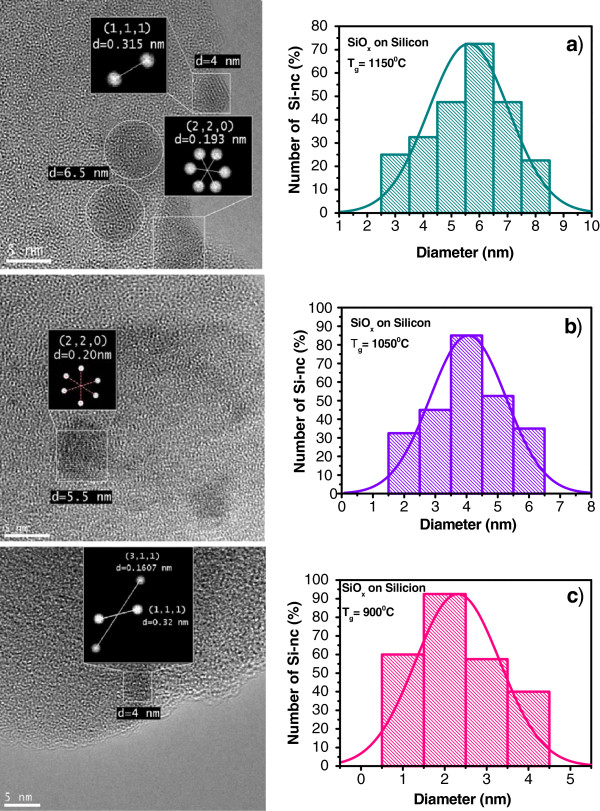
**The plain-view HRTEM images and histograms of the SiO**_***x***_**films.** For samples with Tg at (**a**) 1,150°C, (**b**) 1,050°C, and (**c**) 900°C.

On the other hand, the structure of SiO_*x*_ films was analyzed using the HRTEM technique. Figure 6 shows the HRTEM images for the SiO_*x*_ films, which indicate the presence of Si-ncs embedded in the SiO_*x*_ films. Some of them are semi-elliptical, and some other ones have an enlarged shape. The agglomeration process takes place between the nearest Si-nanoclusters forming Si-nc. All micrographs show that the SiO_*x*_ matrix contains small clusters, which on the basis of the selected area electron diffraction (SAED) analysis can be identified as Si-nc. SAED is referred to as ‘selected’ because, in the micrograph, it can easily choose which part of the sample we obtain the diffraction pattern; in our case, only on the Si-nc. About ten micrographs were obtained to each sample; with them, a statistical analysis of the distribution of the Si-nc diameter sizes was realized. A great dispersion of diameter sizes is observed; the diameter size goes from 1 up to 9 nm, being the average diameter size around 5.5, 4, and 2.5 nm for 1,150°C, 1,050°C, and 900°C, respectively, as indicated in the histograms of Figure 6a,b,c.

From AFM images, the samples grown with lower Tg look more homogeneous than those grown with higher Tg. As shown in the HRTEM images, the silicon excess agglomerates to create Si-ncs; then, the roughness observed in AFM measurements can be associated with Si-ncs and compounds. In addition, FTIR spectra show a phase separation (Si and SiO_2_), which is deduced from the shift of the Si-O stretching vibration mode towards the SiO_2_ frequency value, and it is corroborated with both XPS and HRTEM. Therefore, elemental Si, SiO_*x*_, and SiO_2_ phases with Tg are present, and depending on Si excess, the roughness, size of Si-nc, oxidation states, and vibration modes of the Si-O-Si bonds, some of these phases could be dominant. This indicates that a direct correlation between the roughness, size of Si-nc, oxidation states, and vibration modes of the Si-O-Si bonds exists. In other words, the roughness is produced by the formation of Si-ncs and oxidation states. The diffusion of excess silicon at high Tg produces Si-ncs in the SiO_*x*_ films, i.e., the silicon particles diffuse to create silicon agglomerates around the nucleation sites when the SiO_*x*_ is grown at high Tg.

**Figure 7:**
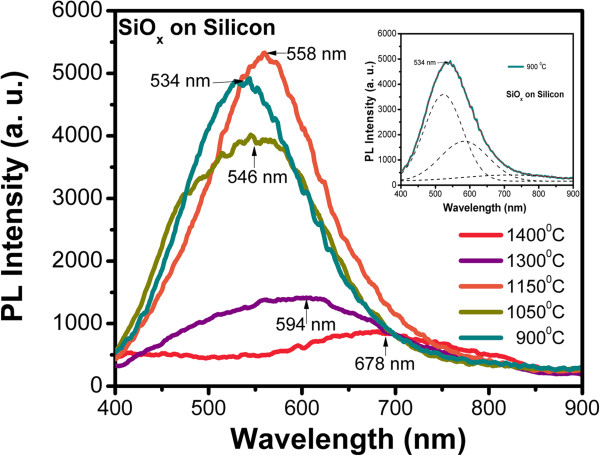
**PL spectra of the SiO**_***x***_**films with different Tg.** Inset shows the convolution realized to PL spectra.

Figure 7 shows the PL response of SiO_*x*_ films corresponding to different growth temperatures. At all samples, a wide PL spectrum is observed. At the growth temperatures of 1,150°C, 1,050°C, and 900°C, the PL peaks are at 558, 546, and 534 nm, respectively. At the highest growth temperature (1,400°C), the PL has the weakest intensity, and the PL peak has the longest wavelength of 678 nm. Moreover, the PL bandwidth and intensity increase for growth temperatures lower than 1,400°C. Therefore, the PL also depends on Tg, size of Si-nc, roughness, oxidation states, and vibration modes of the Si-O-Si bonds, as shown previously.

**Figure 8:**
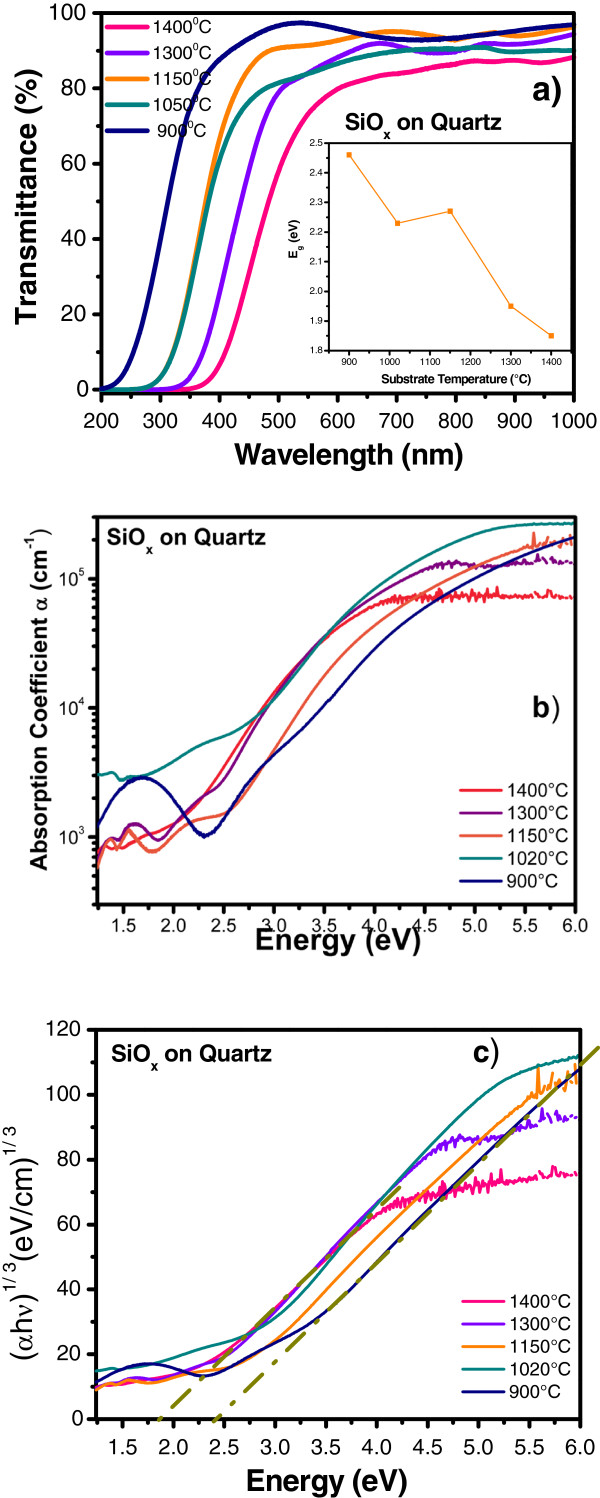
**UV–vis transmittance spectra, absorption coefficient versus energy, and (*αhν*)**^**1/3**^**versus energy (*hν*)*.*** (**a**) UV–vis transmittance spectra and the inset show the energy optical bandgap from SiO_*x*_ films as a function of growth temperature, (**b**) absorption coefficient versus energy, and (**c**) (*αhν*)^1/3^ versus energy (*hν*). The dashed lines show the linear fit of the straight part.

The optical bandgap of SiO_*x*_ films is obtained with transmittance spectra measurements. Transmittance spectra for SiO_*x*_ films deposited on quartz are shown in Figure 8a. The transmittance of all these films is relatively high (>80%) between 600 and 1,000 nm, as shown in the figure, and reduces to zero for wavelengths below 600 nm. The growing temperature produces a clear change of the curves and a shift towards lower wavelengths related to a silicon excess change of the SiO_*x*_ films [11,25,26]. In Tauc’s plot, an increase in the energy bandgap (*E*_g_) has been detected when the growth temperature decreases, as shown in the inset of Figure 8a. The values of the optical bandgap *E*_g_ can be estimated from the following equation known as the Tauc plot [27,28]:

(1) $$\alpha hv=A{\left(hv-E_{g}\right)}^{n}\text{,}$$

where *E*_g_ is the optical bandgap corresponding to a particular transition in the film; *A*, a constant; *ν*, the transmission frequency, which multiplied by the plank constant *h* we have photon energy *hv*, and the exponent *n* characterizes the nature of band transition. The absorption coefficients *α*(*λ*) were determined from transmission spectra with the following relation:

(2) $$\alpha \left(\lambda \right)=\frac{-\text{ln}\left[T\left(\lambda \right)\right]}{d}\text{,}$$

where *T*(*λ*) is the transmittance, and *d* is the thickness of the SiO_*x*_ films. *α* versus *hv* is shown in Figure 8b. On the other hand, values of *n* = 1/2 and 3/2 correspond to direct-allowed and direct-forbidden transitions; *n* = 2 and 3 are related to indirect-allowed and indirect-forbidden transitions, respectively [28]. From a plot (*αhν*)^1/*n*^ versus *hν*, the bandgap can be extrapolated from a straight line to *hν* = 0. For all different growth temperatures, the best straight line is observed for *n* = 3 (Figure 8c), indicating an indirect-forbidden transition mechanism.

The optical properties such as the energy bandgap and the PL bands between (400 to 700 nm) are usually some of the important characteristics of these materials. The PL of SiO_*x*_ films has been extensively studied in the literature [1-25]. Two major mechanisms for PL in this kind of materials are generally accepted: quantum confinement effects in the Si-ncs and defect-related effects, as defects at the Si/SiO_*x*_ interface and defects associated with oxygen vacancies in the film. The first mechanism of light emission that we can consider in the SiO_*x*_ material is related to some kinds of defects produced during the growth process, as shown in the EDS, XPS, and FTIR spectra, where we have bonding such as neutral charged oxygen vacancies (NOV) (Si-Si bonds), non-bridging oxygen hole center (NBOHC), positively charged oxygen vacancies (E’ centers), interstitial oxygen molecules and peroxide radicals [13,14,23,29,30], which can form Si-nps or E’ centers. Therefore, the increase of PL with the Tg is due to the activation of some of these radiative defects. In this study, the 550-nm PL band has been associated with silicon excess in the film in the NOV defects and E’ centers [2,26] types. These bands appear well defined only if the film has been grown with temperatures within 900°C and 1,150°C. If the film was grown at a higher temperature, the band at 700 nm appears with its maximum PL emission.

On the other hand, as a second mechanism of emission, the luminescence peak of SiO_*x*_ films shows a blueshift when the Tg decreases; this behavior is ascribed to quantum confinement effect in the Si-nc. Therefore, in this case the PL spectra are analyzed in terms of a quantum confinement model [1,22]:

(3) $$E_{N}\left(\text{eV}\right)=1,240/\lambda \left(\text{nm}\right)$$

(4) $$E_{N}\left(\text{eV}\right)=1.12\text{eV}+\left(3.73/d^{1.39}\right)\Rightarrow d\left(\text{nm}\right)={\left[\frac{3.73}{E_{N}-1.12}\right]}^{\frac{1}{1.39}}\text{,}$$

which corresponds to the radiative recombination of electron–hole pairs in the Si-nc, where *d* and *E*_N_ are the diameter and energy of the Si-nc, respectively, and *λ* (nm) is the wavelength of the Si-nc emission. Table 3 shows the theoretical values of average size of Si-nc calculated from the PL spectra, where the size of the Si-nc reduces and the energy band gap increases with decreasing the growth temperature, similar to an effect of quantum confinement. Note that unlike as stated in the literature for the quantum confinement effect, PL spectra are very wide which indicates that two possible mechanisms are involved.

**Table 3:** Theoretical values

**Tg (°C)**	**Gap *E***_**N**_**of Si-nc (eV)**		**Diameter of Si-nc (nm)**	
	**Silicon**	**Quartz**	**Silicon**	**Quartz**
*1,400*	1.82	1.87	3.3	3.17
*1,300*	2.08	1.82	2.64	3.3
*1,150*	2.22	2.07	2.4	2.66
*1,050*	2.27	2.16	2.32	2.49
*900*	2.32		2.25	

Values of the *E*_N_ and diameter of the Si-nc calculated from the PL spectra as a function of the Tg.

**Figure 9:**
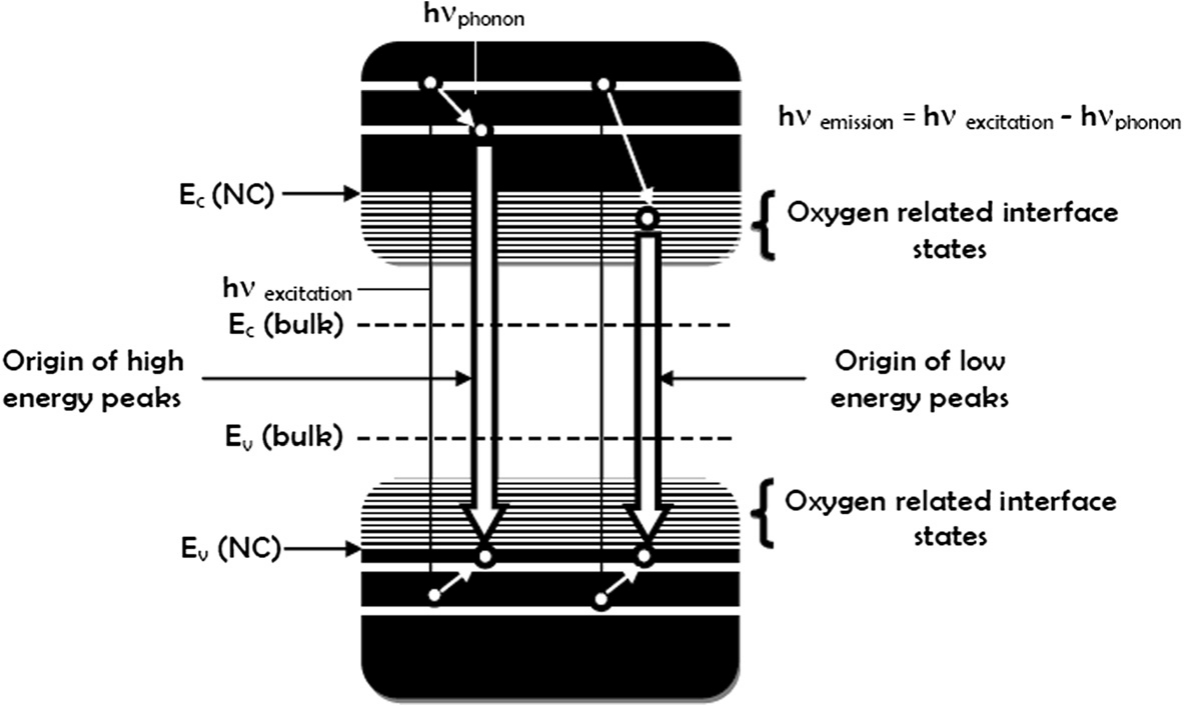
**Schematic representation of the band structure and mechanisms responsible for PL from SiO**_***x***_**films**.

Why is it possible that two mechanisms be involved? When a deconvolution to the PL spectra is made, as shown in the inset of Figure 7, different peaks are defined; some of them are related to different kinds of defects, as listed in Table 4. Therefore, the high-energy PL peaks are associated with quantum confinement effects in Si-ncs, while the low-energy PL peaks are associated with defects. Such a behavior is described in the schematic diagram of the band structure (Figure 9); a similar behavior has been reported previously [31]. This diagram represents the radiative transition giving rise to the emission peaks. For the more energy occurs the generation of electron–hole pairs within the Si-nc core, followed by a thermal relaxation within the conduction band of the Si-nc which in turn suggests the recombination of carriers. In the case of the less energy peaks, phonon relaxation involves more energy because of the transitions between the states of interfacial defects.

**Table 4:** Peak position obtained by deconvolution from PL spectra and defect types relationated with the peak position

**Defect types**	**Peak positions (nm)**					**Reference**
	**1,400°C**	**1,300°C**	**1,150°C**	**1,050°C**	**900°C**	
NOV defects (O_3_ ≡ Si-Si ≡ O_3_)	428	483	458	467		[12]
Centers of defects E’δ				521	523	[12]
E’δ center or oxygen deficiency			553	559		[12,32]
Defect vacancies of oxygen (O ≡ Si-Si ≡ O)		591	600	579	584	[30,33]
Oxide relationated in the interface of Si/SiO_*x*_	675	695				[31,34]
Not identified	796	813				

The existence of Si-ncs in the SiO_*x*_ films was corroborated with the HRTEM measurements. The diffusion of Si excess due to the deposit at high temperature, i.e., when the SiO_*x*_ films are being deposited, could produce Si-ncs. The silicon particles diffuse themselves to create silicon agglomerates around the nucleation sites. If the Si excess is high enough, the Si agglomerates will be crystallized to form Si-ncs. A decrease in the Si-nc diameter has been detected when the growth temperature reduces. The high growth temperature induces the formation of crystals as the statistical analysis of the crystal size distribution, obtained from the HRTEM images, shown. Therefore, the mean diameter of Si-ncs depends on the growth temperature.

Then, two transition mechanisms are possible as the above results and discussion showed widespread bandgap transitions induced by quantum confinement and interface state transitions associated with defects in the oxide. The widespread transitions in Si-nc may bring about high energy peaks (blueshifted PL peaks), and if this energy decays between defects (NOV, NBOHC, and E’ center-related interface states), it can give place to low energy peaks (redshifted PL peaks). All these data indicate that light emission from the films is due to the Si-ncs embedded in the amorphous SiO_*x*_ matrix and defects. Accordingly, we have proposed a combination of mechanisms to explain the photoluminescence in the films.

## Conclusions

SiO_*x*_ films deposited by HFCVD at different growth temperatures were analyzed. These films exhibit an intense PL with a main peak at 550 nm. The strongest PL was obtained for SiO_*x*_ films deposited at 1,150°C. Ellipsometry, XEDS, XPS, FTIR, AFM, PL, transmittance, and HRTEM techniques were used to obtain the structural, compositional, and optical properties of the SiO_*x*_ films, and they were studied as a function of the growth temperature. Thicker samples were obtained when the growth temperature was increased from 900°C to 1,400°C. A variation in the refractive index from 1.4 to 2.2 was obtained when the growth temperature was increased from 1,150°C to 1,400°C. From AFM images, the samples grown with the lower growth temperatures look more homogeneous than those grown with the higher Tg. As shown in the HRTEM images, the silicon excess agglomerates to create Si-ncs; in this way, the roughness observed through AFM measurements can be associated with Si-ncs and compounds. In addition, FTIR spectra show a phase separation (Si and SiO_2_), which is deduced by the shift of the Si-O stretching vibration mode towards the SiO_2_ frequency value, which is corroborated with XPS and HRTEM. A clear relation between the surface roughness, size of Si-nc, oxidation states, composition, and the PL property was obtained. Therefore, PL depends strongly on the Tg and properties of the SiO_*x*_ films.

## Competing interests

The authors declare that they have no competing interests.

## Authors' contributions

JALL and DEVV participated in the growth of the films, carried out the FTIR, PL, and UV measurements and drafted the manuscript. JCL conducted the ellipsometry and AFM measurements. GGS and TDB conducted the SiO_*x*_ growth. APP conducted the HRTEM and XEDS measurements. FJFG coordinated the study. JALL provided the idea and supervised the study. All authors read and approved the final manuscript.

## Authors' information

JALL is currently a researcher and professor in the Science Institute - Center of Investigation in Semiconductors Devices (IC-CIDS) from Autonomous University of Puebla, Mexico. He started to work on electrical and optical characterization of the MOS structures. His research interest is the physics and technology of materials and silicon devices. Moreover, his research interests are, too, the nanotechnology, material characterization, and optoelectronic devices such as sensor, LEDs, and solar cells.

GGS received his PhD in the Electronic and Solid State Department from the Center of Research and Advanced Studies, National Polytechnic Institute, Mexico City in 2003. He started to work on the growth and characterization of non-stoichiometric silicon oxide. His current research interests include metallic oxides obtained by the HFCVD technique, GaN obtained by the metal organic CVD technique and porous silicon gas sensor devices.

FJFG is a coordinator and researcher at the Posgrado en Dispositivos Semiconductores in Benemérita Universidad Autónoma de Puebla, México and was a participant in many important international conferences. Professor FJFG has published many journal articles. His research interests include experiments and models in photoluminescence and quantum confinement in off stoichiometry silicon oxides.
